# Mobility and muscle strength trajectories in old age: the beneficial effect of Mediterranean diet in combination with physical activity and social support

**DOI:** 10.1186/s12966-021-01192-x

**Published:** 2021-09-08

**Authors:** Marguerita Saadeh, Federica Prinelli, Davide L. Vetrano, Weili Xu, Anna-Karin Welmer, Serhiy Dekhtyar, Laura Fratiglioni, Amaia Calderón-Larrañaga

**Affiliations:** 1 Aging Research Center, Department of Neurobiology, Care Sciences and Society, Karolinska Institutet & Stockholm University, Solna, Sweden; 2 SWEAH, Department of Health Sciences, Lund University, Lund, Sweden; 3 Institute of Biomedical Technologies-National Research Council, Milan, Italy; 4 Centro Di Medicina Dell’Invecchiamento, IRCCS Fondazione Policlinico “A. Gemelli” and Catholic University of Rome, Rome, Italy; 5 Functional Area Occupational Therapy & Physiotherapy, Karolinska University Hospital, Stockholm, Sweden; 6 Stockholm Gerontology Research Center, Stockholm, Sweden

**Keywords:** Mobility, Muscle strength, Mediterranean diet, Social support, Physical activity, Older adults

## Abstract

**Introduction:**

Decline in physical function varies substantially across older individuals due to several extrinsic modifiable factors such as dietary patterns, physical activity and social support. We aimed to determine the association of these factors and their interaction with mobility and muscle strength decline.

**Methods:**

We analyzed data from 1686 functionally healthy individuals aged 60 + from the population-based Swedish National study on Aging and Care in Kungsholmen (SNAC-K). The Mediterranean Diet Score (MDS) was calculated based on a validated food frequency questionnaire. Self-reported physical activity was categorized based on current recommendations, and social support was measured according to participants’ perceived material and psychological support from relatives and friends. Participants’ physical function was assessed over 12 years through changes in walking speed (m/s) and chair stand time (s). Linear mixed models adjusted for socio-demographic and clinical factors were used. In order to explore the combined effect of the different exposures, two indicator variables were created by cross-classifying individuals' levels of Mediterranean diet adherence and social support or physical activity.

**Results:**

Participants with a high adherence to Mediterranean diet were primarily < 78 years (82.3%), women (56.1%), married (61.1%), with university education (52.8%), high levels of social support (39.3%) and health-enhancing levels of physical activity (51.5%). A one-point increase in MDS (score range 0–9) was associated with less annual deterioration in walking speed (β*time*[year]* = 0.001; *p* = 0.024) and chair-stand time (β*time*[year]* = -0.014; *p* = 0.008). The potential protective effect of Mediterranean diet was highest among participants reporting high social support (β*time*[year]* = -0.065, *p* = 0.026 for chair stands) and high physical activity (β*time*[year]* = 0.010, *p* = 0.001 for walking speed), beyond the effect of each exposure individually.

**Conclusion:**

A higher adherence to Mediterranean diet, especially in combination with recommended levels of physical activity and high social support, may contribute to delay the decline in physical function observed with aging.

**Supplementary Information:**

The online version contains supplementary material available at 10.1186/s12966-021-01192-x.

## Introduction

Adding life to the increasing number of years lived by older people has become a major goal of modern geriatric medicine [1]. A healthy dietary pattern and high levels of physical activity and social support characterize the inhabitants of the so-called Blue Zones of North America, Japan, Costa Rica, Greece and Sardinia, where people live much longer and healthier than average [2]. Indeed, according to the revisited version of Rowe and Kahn’s model of successful aging, behavioral factors together with psychosocial well-being have been highlighted as the main determinants of preserved functionality in old age [1, 3]. Consistently across the literature, mobility and muscle strength have been identified as optimal indicators of physical function [4].

The Mediterranean diet (MD) is considered a healthy dietary pattern for its contribution to a favorable overall health status [5]. It is characterized by high intake of vegetables, fruits, legumes, cereals, fish and unsaturated lipids (e.g., olive oil); a moderate intake of meat, dairy products and a low intake of saturated lipids and ethanol, mostly as red wine [6]. A beneficial role of its main components in delaying the onset of physical impairment, functional disability and muscle health has been reported [7]. Only recently, studies looking at the role of MD on muscle aging and its potential to protect muscle tissue from damage (i.e., *myoprotection*) have emerged [8, 9]. The number of studies is even smaller in non-Mediterranean European countries, such as Sweden, where food composition could vary due to differences in seasonal food availability and dietary habits. According to a community-based study in older Swedish men (mean age 71 years), 13% of the study participants reported having high adherence to MD [10].

The protective effect of physical activity on physical frailty, a clinically recognizable state of increased vulnerability resulting from aging-associated decline in reserve and function across multiple physiologic systems, has been extensively demonstrated [11]. It is also a protective factor for several chronic and metabolic diseases such as cardiovascular diseases and diabetes [12], and has been associated with better mental health and delayed onset of dementia [13]. Physical activity levels amongst Swedish older adults remain below official recommendations, despite its acknowledged health benefits [14].

Social support is another feature of successful aging whereby older adults may engage in collective activities and establish diverse social relationships [15]. Having a high social support has been inversely associated with frailty [16]. These associations are partly explained by the role of social support in reducing the harmful effects of both chronic and short-term stress [17]. Older people with high social support also show greater adherence to healthier patterns of diet [18, 19] and physical activity [20], leading to a possible positive feedback loop between these three components. In one of our previous studies, we showed that 50% of healthy participants older than 60 years living in central Stockholm reported low levels of social support, connections and participation [21].

Despite the growing amount of literature on the added value of a multifactorial approach to preserve physical function in old age, very few studies have looked at the synergistic effect of MD, together with physical activity and social support in community-dwelling healthy populations. Moreover, provided the limited access to longitudinal data, previous research has rarely examined physical function as trajectories of both mobility and muscle strength, despite their acknowledged association with major health endpoints including frailty [22] and sarcopenia [23], i.e., the age-related loss of skeletal muscle mass and function. The aim of our study was to explore the association between MD, in combination with physical activity and social support, and the rate of decline in mobility and strength in Swedish older adults.

## Materials and Methods

### Study population

Data from the Swedish National study on Aging and Care in Kungsholmen (SNAC-K; http://www.snac-k.se) were used for this study. The ultimate goal of SNAC-K is to better understand the aging process, and to identify possible preventive strategies to improve health and care in older adults. This is an ongoing community-based longitudinal study of randomly selected adults aged 60 years or older living at home or in institutions in the Kungsholmen district of Stockholm between 2001 and 2004. After stratifying the target population in 11 age cohorts (ages 60, 66, 72, 78, 81, 84, 87, 90, 93, 96 and ≥ 99 years), a random sample of 4590 individuals was selected. The baseline SNAC-K population included 3363 individuals (73.3% participation rate) who were aged between 60–104 years at baseline and have been followed up regularly: every six years for the young-old cohorts (< 78 years) and every three years for the older cohorts (≥ 78 years). Trained physicians, nurses, and psychologists conducted extensive clinical examinations, interviews and assessments on participants following the same protocols in all study waves. SNAC-K data is linked to the National Patient Register and the Swedish Cause of Death Register to obtain information on medical history and vital status.

This study included data from SNAC-K baseline and first four follow-ups covering up to 12 years. In order to minimize reverse causality, we first excluded 1307 individuals (38.9%) with walking speed < 0.8 meters per second (m/s) or five chair stands > 17 seconds (s) at baseline according to established cutoffs [24, 25] (Additional file 1). We further excluded 86 participants (4.2% of the remaining sample) with a definite or questionable dementia diagnosis according to the Diagnostic and Statistical Manual of Mental Disorders (4^th^ edition), and 18 participants (0.9% of the remaining sample) with a Mini-Mental State Exam (MMSE) score < 24 (both at baseline) because of the limited reliability of their self-reported exposures. We also excluded 260 individuals (13.3% of the remaining sample) with > 20% missing data on diet and 6 individuals (0.3% of the remaining sample) with missing data on social support (both at baseline). After applying all exclusion criteria, 1686 participants (86.4% of the eligible population) remained in the study sample (Additional file 1). SNAC-K was approved by the Regional Ethical Review Board in Stockholm, and written informed consent was obtained from participants or their next of kin at each follow-up wave.

### Mediterranean diet

Usual dietary intakes were assessed at baseline using a validated self-administered 98-item semi-quantitative food frequency questionnaire (SFFQ) [26]. Participants were asked to report their average intake frequency of each food and beverage over the past 12 months on a fixed 9-level scale ranging from never to ≥ 4 times per day. Zero imputation was applied to replace SFFQ missing items (Additional file 2). Portion sizes of staple foods (potatoes, rice, and pasta), meat/fish, and vegetables were estimated by providing color photos of four plates with increasing portion sizes in the SFFQ. Standard portion sizes (e.g., the size of an average apple as one portion of fruit) were used for other food items. Frequencies were converted into daily consumption and the energy intake was calculated by multiplying these frequencies by a portion size value and by the energy content, based on a food composition database from the National Food Administration using MATs software Version 4.03 (Rudans Lättdata, Västerås, Sweden) [27].

A score indicating the degree of adherence to MD was calculated according to the formula proposed by Trichopoulou et al. [6]. Each of the nine indicated components were assigned a value of zero or one, using the sex specific medians as cut-offs. People whose consumption of presumed beneficial components (i.e., vegetables, legumes, fruits, cereals, fish) was below the median were assigned a value of zero, and a value of one otherwise. People whose consumption of potentially less healthful components (i.e., meat and dairy products) was below the median were assigned a value of one, and a value of zero otherwise. For alcohol intake, men consuming from 10 g to less than 50 g per day and women consuming from 5 to 25 g were given a value of one. The food composition, as well as food production and preparation, can differ significantly between the Mediterranean and non-Mediterranean populations. These differences may modify the potential protective effects of MD in non-Mediterranean countries. The main difference is related to the consumption of monounsaturated fatty acids from olive oil, which is considered minimal in non-Mediterranean populations. Therefore, fat intake was calculated through the ratio of the sum of monounsaturated (MUFA) and polyunsaturated (PUFA) to saturated (SFA) fatty acids, as proposed in the modified MD score (MDS) [6]. The dietary components and the cut-offs used for men and women to create the MDS in SNAC-K are shown in Additional file 2. The score was adjusted for total energy intake using the residual method [28]. The MDS could take values from zero (minimal adherence) to nine (maximal adherence), and was subsequently categorized into tertiles as low, moderate and high adherence.

### Social support and physical activity

Social support was measured by asking participants about their satisfaction, perceived material and psychological support from parents, children, relatives, neighbors and friends, and their sense of kinship with association members, relatives, neighborhood and friends. The social support variable was created after standardizing and averaging all previously mentioned items, and was subsequently categorized according to tertiles as low vs high levels (moderate/high) [21]. Information on the intensity and frequency of physical activity was obtained through a self-administered questionnaire. The intensity was defined as light exercise (walks on the sidewalk or paved surfaces, in parks, in forests, short bike rides, light aerobic activities or gym classes, golf) and moderate to intense exercise (brisk walking, jogging, heavy gardening, long bike rides, intense aerobic activities or gym classes, skating, skiing, swimming, ball games or similar activities). Following the recommendations of the World Health Organization (WHO) and the American College of Sports Medicine (ACSM), participants were classified into three levels of physical activity: inadequate (≤ 2–3 times per month of light and/or moderate/intense exercise), health-enhancing (light exercise several times per week or every day), and fitness-enhancing (moderate/intense exercise several times per week or every day) [29]. We further dichotomized physical activity into low levels (inadequate and health-enhancing) and high levels (fitness-enhancing), according to the distribution of the variable in our study population.

### Mobility and strength

Walking speed was assessed by trained nurses whereby participants were asked to walk 6 m or 2.4 m, at a self-selected speed and using a walking aid if needed. The length of the walk was determined by asking participants how fast they normally walked (i.e., normal/fast walkers did the longer walk and slow/very slow walkers did the shorter walk). It was reported as meters per seconds (m/s) reflecting the speed for whichever length walked. Chair stands were tested by asking participants to fold their arms across their chest and stand up from a seated position five times consecutively as quickly as possible, and the results were expressed in seconds. Higher walking speed (m/s) and lower chair stands (s) scores indicate better mobility and strength, respectively. Participants with severe physical limitations and those who were unable to perform any of the lower extremity tests received the worst possible score, i.e., a walking speed of 0 m/s and a 75-s chair stand time.

### Covariates

Several covariates were considered as possible confounders and were measured at baseline: age, sex, highest level of formal education (elementary school, high-school, or university and above), number of chronic conditions defined as those with prolonged duration and either (a) leaving residual disability or worsening quality of life or (b) requiring a long period of care, treatment, or rehabilitation [30], civil status (unmarried/married/divorced/widow), and dietary supplements use (yes/no) including vitamin A, B1, B6, B12, C, other plain vitamins, calcium, potassium and other minerals. Time to death and time to dropout were measured using data from all follow-up waves (Additional file 1).

### Statistical analysis

Linear mixed models were used to estimate β-coefficients and 95% confidence intervals for the association between baseline adherence to MD and annual changes in walking speed and chair stands over the 12-year follow-up. An interaction term between follow-up time and MD was included as a fixed effect. For walking speed, a positive β-coefficient for the interaction indicates that a higher adherence to MD is associated with a slower decline, while for chair stands, a negative β-coefficient indicates a slower decline over time. Models were first adjusted for sex, age and education level (Model I), and additionally for civil status, number of chronic diseases, use of dietary supplements, survival and dropout statuses (Model II). The exposure was operationalized both as continuous and categorized into tertiles (low MDS = 0–4; moderate MDS = 5; high MDS = 6–9) in order to address potential non-linearity in the associations and to facilitate the interpretation of the findings. Three-way interactions between MDS*social support*time and MDS*physical activity*time were also tested. We created two indicator variables with four mutually exclusive categories by cross-classifying individuals' levels of MD adherence (low MDS = 0–4; high MDS = 5–9, according to the median) and social support (low; moderate/high) or physical activity (low; high) in order to further explore the combined effect of the three exposures. As sensitivity analyses, we reran the models: a) adjusting additionally for physical activity and social support to examine the independent association of MDS with walking speed and chair stand trajectories; b) after excluding participants with less than two measures of physical function, in order to study the impact of longitudinal attrition; and c) after excluding participants with missing data on any individual food item comprising the MDS (i.e., complete case analysis). All analyses were performed using Stata version 15 with the level of statistical significance set at *p* < 0.05. An extension of the STROBE statement for nutritional epidemiology was used for reporting this study (Additional file 3).

## Results

At baseline, the study population consisted of 1686 individuals, 58% being female and with a mean (standard deviation, SD) age of 69 (8.1) years (age range 60–94 years). The majority had at least a high-school level education (91%), was married (55.5%) and, performed health-enhancing levels of physical activity (53.9%). Almost 48% of the participants had a low adherence to MD (Table 1). Older men and women reported lower levels of social support compared to younger participants. Those with a high adherence to MD were primarily < 78 years (82.3%), women (56.1%), married (61.1%), with university education (52.8%), high levels of social support (39.3%) and health-enhancing levels of physical activity (51.5%) (Table 2). Excluded participants were older, had more chronic diseases, reported lower adherence to MD, lower social support, inadequate/health-enhancing physical activity levels, and had a slower walking speed and longer chair stand time (Additional file 4).

**Table 1:** Baseline sociodemographic, clinical and lifestyle characteristics of the study population by age and sex (*N* = 1686)

	**Total population**	** < 78 years (n = 1314)**		** ≥ 78 years (n = 372)**	
		**Male (n = 573)**	**Female (n = 741)**	**Male (n = 141)**	**Female (n = 231)**
**Education, n(%)**					
Elementary	152 (9.0)	46 (8.0)	49 (6.6)	22 (15.6)	35 (15.2)
High school	784 (46.5)	225 (39.3)	344 (46.4)	67 (47.2)	148 (64.1)
University	750 (44.5)	302 (52.7)	348 (47.0)	52 (36.9)	48 (20.8)
**Civil status, n(%)**					
Unmarried	275 (16.3)	104 (18.2)	134 (18.1)	10 (7.1)	27 (11.7)
Married	935 (55.5)	396 (69.1)	380 (51.4)	100 (70.9)	59 (25.5)
Divorced	237 (14.1)	56 (9.8)	146 (19.8)	8 (5.7)	27 (11.7)
Widow	237 (14.1)	17 (3.0)	79 (10.7)	23 (16.3)	118 (51.1)
**Chronic diseases, median (IQR)**	3.1 (1.7;2.9)	2 (1;4)	3 (2;4)	4 (3;6)	4 (3;6)
**Dietary supplements, n(%)**					
No	1262 (74.9)	507 (88.5)	517 (69.8)	109 (77.3)	129 (55.8)
Yes	424 (25.2)	66 (11.5)	224 (30.2)	32 (22.7)	102 (44.2)
**Social support, n(%)**					
Low	562 (33.3)	197 (34.4)	208 (28.1)	74 (52.5)	83 (35.9)
Moderate	562 (33.3)	191 (33.3)	257 (34.7)	35 (24.8)	79 (34.2)
High	562 (33.3)	185 (32.3)	276 (37.3)	32 (22.7)	69 (29.9)
**Physical activity, n(%)**					
Inadequate	263 (15.6)	109 (19.0)	92 (12.4)	22 (15.6)	40 (17.3)
Health enhancing	908 (53.9)	270 (47.1)	410 (55.3)	82 (58.2)	146 (63.2)
Fitness enhancing	515 (30.6)	194 (33.9)	239 (32.3)	37 (26.2)	45 (19.5)
**Adherence to Mediterranean diet, n(%)**					
Low	802 (47.6)	260 (45.4)	338 (45.6)	66 (46.8)	138 (59.7)
Moderate	365 (21.7)	129 (22.5)	160 (21.6)	31 (22.0)	45 (19.5)
High	519 (30.8)	184 (32.1)	243 (32.8)	44 (31.2)	48 (20.8)
**Total energy intake (Kcal/day),** **median (IQR)**	1601.6 (1274.6;1992.7)	1824.9 (1469.9;2190.7)	1443.8 (1172.0;1741.5)	2040.5 (1644.7;2379.9)	1442.1 (1165.8;1818.8)

Dietary supplements include vitamin A, B1, B6, B12, C, other plain vitamins, calcium, potassium and other minerals

Low, moderate and high levels of social support and adherence to Mediterranean diet categorized according to the tertiles of the distribution

Levels of physical activity categorized as follows: inadequate (≤ 2–3 times per month of light and/or moderate/intense exercise), health-enhancing (light exercise several times per week or every day), and fitness-enhancing (moderate/intense exercise several times per week or every day)

*IQR* Interquartile range

**Table 2:** Baseline sociodemographic, clinical and lifestyle characteristics of the study population by levels of adherence to Mediterranean diet (N = 1686)

	**Low adherence (n = 802)**	**Moderate adherence (n = 365)**	**High adherence (n = 519)**	*p*-value*
**Age n(%)**				
< 78 years	598 (74.6)	289 (79.2)	427 (82.3)	** < 0.001**
≥ 78 years	204 (25.4)	76 (20.8)	92 (17.7)	
**Sex n(%)**				
Male	326 (40.7)	160 (43.8)	228 (43.9)	**0.011**
Female	476 (59.4)	205 (56.2)	291 (56.1)	
**Education, n(%)**				
Elementary	92 (11.5)	26 (7.1)	34 (6.6)	** < 0.001**
High school	396 (49.4)	177 (48.5)	211 (40.7)	
University	314 (39.2)	162 (44.4)	274 (52.8)	
**Civil status, n(%)**				
Unmarried	154 (19.2)	45 (12.3)	76 (14.7)	** < 0.001**
Married	405 (50.5)	214 (58.6)	316 (61.1)	
Divorced	115 (14.3)	59 (16.2)	63 (12.2)	
Widow	128 (16.0)	47 (12.9)	62 (12.0)	
**Chronic diseases, median (IQR)**	3 (2;4)	3 (2;4)	3 (2;4)	** < 0.001**
**Dietary supplements, n(%)**				
No	596 (74.3)	279 (76.4)	387 (74.6)	0.205
Yes	206 (25.7)	86 (23.6)	132 (25.4)	
**Social support, n(%)**				
Low	296 (36.9)	115 (31.5)	151 (29.1)	** < 0.001**
Moderate	281 (35.0)	117 (32.1)	164 (31.6)	
High	225 (28.1)	133 (36.4)	204 (39.3)	
**Physical activity, n(%)**				
Inadequate	150 (18.7)	53 (14.5)	60 (11.6)	** < 0.001**
Health enhancing	442 (55.1)	199 (54.5)	267 (51.5)	
Fitness enhancing	210 (26.2)	113 (30.9)	192 (37.0)	
**Total energy intake (Kcal/day),** **median (IQR)**	1571.5 (1216.3;2013.8)	1610.9 (1316.6;1943.9)	1628.4 (1327.0;2008.1)	** < 0.001**

^*^Chi2 test or Kruskall-Wallis test accordingly

Low, moderate and high levels of social support and adherence to Mediterranean diet categorized according to the tertiles of the distribution

Levels of physical activity categorized as follows: inadequate (≤ 2–3 times per month of light and/or moderate/intense exercise), health-enhancing (light exercise several times per week or every day), and fitness-enhancing (moderate/intense exercise several times per week or every day)

*IQR* Interquartile range

**Fig. 1:**
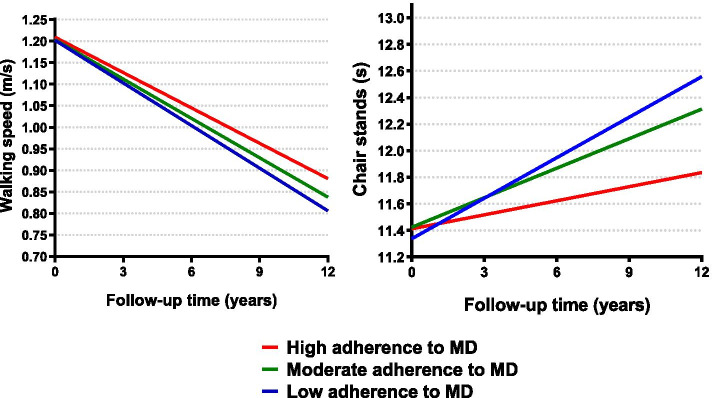
Estimated walking speed (m/s) and chair stands (s) over the 12-year follow-up by levels of Mediterranean diet adherence (N = 1686). Low, moderate and high levels of adherence to Mediterranean diet categorized according to the tertiles of the distribution. MD: Mediterranean diet

**Fig. 2:**
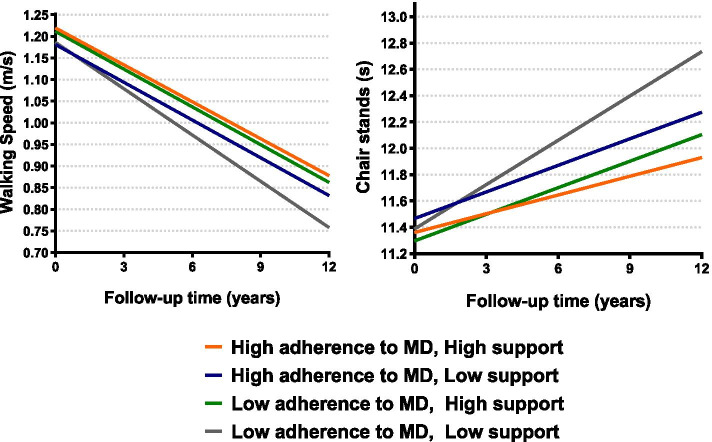
Estimated walking speed (m/s) and chair stands (s) over the 12-year follow-up by levels of Mediterranean diet adherence and social support (N = 1686). Low and high levels of adherence to Mediterranean diet categorized according to the median of the distribution; and low and high (i.e., moderate/high) levels of social support according to the tertiles of the distribution. MD: Mediterranean diet

**Fig. 3:**
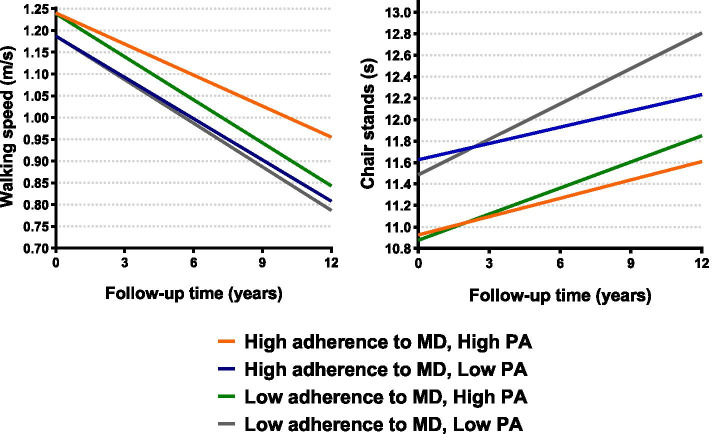
Estimated walking speed (m/s) and chair stands (s) over the 12-year follow-up by levels of Mediterranean diet adherence and physical activity (N = 1686). Low and high levels of adherence to Mediterranean diet categorized according to the median of the distribution, and low (i.e., inadequate and health-enhancing) and high (i.e., fitness-enhancing) levels of physical activity according to official recommendation, as described in the methods. MD: Mediterranean diet; PA: physical activity

In the longitudinal analyses over the 12-year follow-up, a one-point (equivalent to 11%) increase in the MDS was associated with a slower worsening in walking speed (β*time*[year]* = 0.001; *p* = 0.024) and chair stand time (β*time*[year]* = -0.014; *p* = 0.008), after adjusting for potential confounders (Table 3, Fig. 1). Results remained similar after additionally adjusting for physical activity and social support (Additional file 5), after excluding participants with less than two measures of physical function (Additional file 6), and when carrying the complete case analysis (Additional file 7). According to the combined indicator variables, those with high levels in both adherence to MD and social support showed the highest protective effect (beyond the effect of each exposure individually), especially for the chair stand test (β*time*[year]* = -0.065, *p* = 0.026 for high/high vs low/low categories) (Fig. 2, Additional file 8). In addition, those with high levels in both adherence to MD and physical activity showed the slowest decline in walking speed compared to the other groups, beyond the independent protective effect of both exposures (β*time*[year]* = 0.010, *p* = 0.001 for high/high vs low/low categories). This was less evident for the chair stand test, where people with high levels of MD but low levels of physical activity showed similar rates of decline. (Fig. 3, Additional file 8). Three-way interactions between MDS*social support*time and MDS*physical activity*time were not statistically significant (Additional file 8, footnotes).

**Table 3:** Association between adherence to Mediterranean diet and annual decline in walking speed (m/s) and chair stands (s) over the 12-year follow-up (N = 1686)

	**Model I**		**Model II**	
	**β (95% CI)**	***p*-value**	**β (95% CI)**	***p*-value**
**Walking speed (m/s)**				
**Continuous**	0.001 (0.0001;0.002)	**0.035**	0.001 (0.0002;0.002)	**0.024**
**Categorical**				
Low	Ref	Ref	Ref	Ref
Moderate	0.002 (-0.003;0.007)	0.446	0.002 (-0.003;0.007)	0.347
High	0.005 (0.001;0.010)	**0.020**	0.005 (0.001; 0.010)	**0.013**
**Chair stands (s)**				
**Continuous**	-0.014 (-0.025;-0.003)	**0.009**	-0.014 (-0.025; -0.004)	**0.008**
**Categorical**				
Low	Ref	Ref	Ref	Ref
Moderate	-0.021 (-0.068;0.026)	0.381	-0.023 (-0.070;0.024)	0.343
High	-0.063 (-0.105;-0.022)	**0.003**	-0.064 (-0.105;-0.022)	**0.003**

Model I: adjusted by sex, age, education level

Model II: adjusted additionally by civil status, number chronic diseases at baseline, dietary supplements and death/dropouts

Low, moderate and high levels of adherence to Mediterranean diet categorized according to the tertiles of the distribution. *CI* Confidence interval

## Discussion

Using data from a Swedish population-based study of older adults aged 60 years and above, we found that a higher adherence to MD was independently associated with slower decline in walking speed and a slower increase in the time needed for the chair stand test over a 12-year follow-up. We also found that having high levels of social support and physical activity, two major components underlying successful aging, significantly contributed to strengthen the associations, suggesting a potential synergy among exposures. Even if effect sizes were relatively small in clinical terms, one should bear in mind that numbers result from multivariate models performed in functionally healthy older adults; the impact of our exposures is thus likely to be underestimated.

Despite the growing literature on dietary patterns and physical function, very few studies have looked at the longitudinal association between MD and mobility or strength in older adults, especially in non-Mediterranean areas. Still, the existing evidence supports our findings. Two studies based on the InCHIANTI cohort of older men and women living in Tuscany, Italy, showed that a higher adherence to MD was associated with a slower decline in mobility over nine years of follow-up [31], as well as with a lower odds of developing frailty, low physical activity and slow walking speed after six years [32]. However, no association was found with muscle strength measured through grip strength [32]. The Health, Aging and Body Composition Study (Health ABC) including a racially diverse sample from the US showed that over eight years of follow-up, older individuals aged > 70 years with a high adherence to MD had significantly faster walking speed tests [33]. Similarly, two studies based on the Seniors-ENRICA cohort of Spanish participants aged ≥ 60 years showed that a Mediterranean-style dietary pattern was associated with a lower likelihood of physical function impairment [34], walking speed decline, weight loss and frailty development in older adults [35]. Another longitudinal French study from the Three-City-Bordeaux center based on non-frail participants showed a 68% frailty risk reduction, defined as incident slowness, poor muscle strength and low physical activity, among those with the highest versus lowest adherence to MD after two years of follow-up [36].

Mediterranean Diet is characterized by a higher intake of bioactive compounds (i.e., vitamins, minerals, mono- and polyunsaturated fatty acids, protein) and specific foods (i.e., vegetables, fruits, legumes, whole grains, olives/vegetable oils) that exert important health benefits [37]. MD’s antioxidant properties play an important role in the preservation of physical function, given the primary role of oxidative stress in muscle function and health [38]. Oxidative stress, elicited by the accumulation of reactive oxygen and nitrogen species, damages the structure and function of basic structural components (i.e., proteins, lipids and nucleic acid). When endogenous antioxidant enzymes (e.g., glutathione peroxidase and catalase, thioredoxin, etc.) fail in neutralizing the damaging effects of excess free radicals in myofibers, the intake of exogenous antioxidants via an antioxidant-rich diet such as MD becomes crucial in preserving the neuromuscular function [38–40]. The anti-inflammatory properties of MD, through its effects on pro-inflammatory cytokines (i.e., IL-6, IL-8 and TNF-α), have also been shown to alleviate age-related loss of muscle mass and function [40]. MD is considered to be less-acidic than other dietary patterns, which possibly reduces muscle wasting and sarcopenia, conferring further myoprotection [38].

Besides its effect on the preservation of muscle mass, and therefore of muscle strength [41], MD may also prevent mobility decline through its beneficial effect on the central and peripheral nervous systems, the cardiovascular system, and the metabolic system [42]. It has been shown to prevent the occurrence of neurodegenerative diseases [43] and brain vascular damage [44], most likely by lowering the levels of amyloid-β in key brain areas [45], and by increasing the levels of progenitor cells that protect against the development of endothelial dysfunction and cerebral hypoperfusion [46]. Furthermore, a high adherence to MD has shown to reduce the rates of ischemic stroke and cardiovascular diseases by increasing HDL levels, promoting antioxidant effects and reducing inflammation and systemic hypoperfusion [47]. MD and its main components are also negatively associated with insulin resistance and type 2 diabetes [48], which further contributes to preserve muscle health [9].

Mediterranean Diet alone cannot explain the complex etiology of chronic degenerative diseases and functional impairment, and their prevention requires multifactorial approaches focusing on other key extrinsic modifiable risk factors such as physical activity and social support [15]. The Survey on Health, Ageing and Retirement in Europe (SHARE) including 22,226 community-dwelling robust and pre-frail European older adults is one of the few studies that looked at these different risk factors comprehensively [49]. The authors concluded that older individuals with a combination of two out of the three studied risk factors (i.e., low protein intake, decreased physical activity and weak social network) had a higher risk for frailty compared to those with no or one risk factor. Indeed, dietary and nutritional treatment and physical activity constitute fundamental aspects of the multidimensional preventive and therapeutic approach proper of geriatric medicine [50]. Further, it has been shown that increased physical activity together with a high adherence to MD is associated with a multiplicative rise in total antioxidant capacity levels of our organism [51]. Physical activity seems to also directly influence the body composition through increased protein uptake and microfibrillar protein synthesis [52], vascularization and energy metabolism [53]. Furthermore, it prevents the decline in mitochondrial respiration, mitigating aging-related loss of muscle mass and enhancing insulin sensitivity [54].

Fewer studies have looked at plausible mechanisms explaining the effect of social support on physical function. Social isolation and lack of social support have been associated with worse diet quality [18] and lower fruit and vegetable consumption in older adults, possibly due to the lack of help from family and friends with shopping and cooking chores [55]. Eating is also a social activity and doing it in company will determine the quality and quantity of food that is eaten [19]. In a study of 238 homebound older adults receiving home health services, support provided once a day by a daughter-caregiver reduced the risk of malnutrition and highlighted the importance of commensality, defined as the act of eating together [56]. Perceived support increases older individuals’ coping capacity, helps them downplay the age-related problems faced, and thus maintain healthy habits and better diet [19]. The stress-buffering effect of social support is also directly linked to a better physical and mental health [57], possibly by preventing inflammatory processes [17], among others.

Strengths of this study include its longitudinal design with a long follow-up and a large sample of community-dwelling older adults. Moreover, we used objective measures of walking speed and chair stands assessed by qualified healthcare professionals at multiple time points, providing us with reliable information on their temporal evolution. Last, we employed the modified MDS, which is better suited to a non-Mediterranean population like our study population of Swedish older adults. However, some limitations need to be acknowledged. The study sample included healthy and relatively wealthy participants who were able to self-report their health behaviors, which limits the generalizability of our results to the SNAC-K population and probably its transferability to other cohorts of older adults. Moreover, all three exposures were self-reported, which may have introduced some measurement error. Also, the zero imputation carried out for the missing food items within the SFFQ might have led to misclassification of the intake; however, in dietary analyses it is typically assumed that missing values in the questionnaire most often reflects that the food was not consumed [58]. Despite excluding participants with impaired mobility or strength at baseline and adjusting our models for several confounders, reverse causality and/or residual confounding may not be fully discarded. In addition, other aspects of social well-being (e.g., neighborhood social cohesion) were not explored. However, we believe that they are most likely reflected in the individual-level social support measure included in our study. Finally, the lack of repeated measures over time for MD, physical activity and social support could be problematic among participants who are less likely to maintain similar behaviors over time.

## Conclusion

In summary, diet, physical activity and social support are modifiable factors amenable to interventions that could potentially improve the primary prevention of accelerated decline in mobility and muscle strength among community-dwelling older adults. Further longitudinal studies with detailed clinical and biological data are needed to confirm our results, and to better understand the mechanisms through which the synergies between these different exposures buffer the decline in physical function. This knowledge will be an important step towards designing effective holistic interventions to promote successful aging.

## Supplementary Information

**Additional file 1**. Population flow-chart for baseline and follow-up assessments.

**Additional file 2**. Dietary components of MDS and their cut-offs according to the median by sex.

**Additional file 3**. STROBE-nut: An extension of the STROBE statement for nutritional epidemiology.

**Additional file 4**. Baseline sociodemographic, clinical and lifestyle characteristics of the included and excluded population.

**Additional file 5**. Sensitivity analysis. Association between adherence to Mediterranean diet and annual decline in walking speed (m/s) and chair stands (s) over the 12-year follow-up (N=1686). Results after adjusting additionally by physical activity and social support.

**Additional file 6**. Sensitivity analysis. Association between adherence to Mediterranean diet and annual decline in walking speed (m/s) and chair stands (s) over the 12-year follow-up. Results after excluding participants with less than two measures of walking speed (m/s) or chair stands (s).

**Additional file 7**. Sensitivity analysis. Association between adherence to Mediterranean diet and annual decline in walking speed (m/s) and chair stands (s) over the 12-year follow-up (N=892). Results after excluding participants with any missing data on the food items comprising the MDS (i.e. complete case analysis).

**Additional file 8**. Association between levels of adherence to Mediterranean diet and annual decline in walking speed (m/s) and chair stands (seconds) over the 12-year follow-up, by levels of social support and physical activity (N=1686).

## Data Availability

Data are from the SNAC-K project, a population-based study on aging and dementia (http://www.snac-k.se/). Access to these original data is available to the research community upon approval by the SNAC-K data management and maintenance committee. Applications for accessing these data can be submitted to Maria Wahlberg (Maria.Wahlberg@ki.se) at the Aging Research Center, Karolinska Institutet.

## References

[1] Michel J-P, Sadana R (2017). “Healthy aging” concepts and measures. J Am Med Dir Assoc.

[2] Buettner D, Skemp S (2016). Blue Zones. Am J Lifestyle Med.

[3] Sabia S, Singh-Manoux A, Hagger-Johnson G, Cambois E, Brunner EJ, Kivimaki M (2012). Influence of individual and combined healthy behaviours on successful aging. Can Med Assoc J.

[4] Anton SD, Woods AJ, Ashizawa T, Barb D, Buford TW, Carter CS (2015). Successful aging: advancing the science of physical independence in older adults. Ageing Res Rev.

[5] Godos J, Castellano S, Marranzano M. Adherence to a mediterranean dietary pattern is associated with higher quality of life in a cohort of Italian adults. Nutrients. 2019;11(5):981. 10.3390/nu11050981 PMC6566890 31035736

[6] Trichopoulou A, Orfanos P, Norat T, Bueno-de-Mesquita B, Ocké MC, Peeters PHM (2005). Modified Mediterranean diet and survival: EPIC-elderly prospective cohort study. Br Med J.

[7] Gielen E, Beckwée D, Delaere A, De Breucker S, Vandewoude M, Bautmans I, et al. Nutritional interventions to improve muscle mass, muscle strength, and physical performance in older people: an umbrella review of systematic reviews and meta-analyses. Nutr Rev. 2021;79(2):121–47. 10.1093/nutrit/nuaa011 32483625

[8] Bloom I, Shand C, Cooper C, Robinson S, Baird J (2018). Diet quality and sarcopenia in older adults: a systematic review. Nutrients.

[9] McClure R, Villani A. Mediterranean Diet attenuates risk of frailty and sarcopenia: New insights and future directions. JCSM Clin Rep. 2017;2(2):1–17.

[10] Franzon K, Byberg L, Sjögren P, Zethelius B, Cederholm T, Kilander L (2017). Predictors of Independent aging and survival: a 16-year follow-up report in octogenarian men. J Am Geriatr Soc.

[11] Haider S, Grabovac I, Dorner TE. Fulfillment of physical activity guidelines in the general population and frailty status in the elderly population: A correlation study of data from 11 European countries. Wien Klin Wochenschr. 2019;131(11-12):288–93. 10.1007/s00508-018-1408-y PMC6570679 30421283

[12] Marques A, Peralta M, Martins J, de Matos MG, Brownson RC (2017). Cross-sectional and prospective relationship between physical activity and chronic diseases in European older adults. Int J Public Health.

[13] Iuliano E, di Cagno A, Cristofano A, Angiolillo A, D’Aversa R, Ciccotelli S (2019). Physical exercise for prevention of dementia (EPD) study: background, design and methods. BMC Public Health.

[14] Rydwik E, Welmer A-K, Kåreholt I, Angleman S, Fratiglioni L, Wang H-X (2013). Adherence to physical exercise recommendations in people over 65–the SNAC-Kungsholmen study. Eur J Public Health.

[15] Diolintzi A, Panagiotakos DB, Sidossis LS (2019). From Mediterranean diet to Mediterranean lifestyle: a narrative review. Public Health Nutr.

[16] De Labra C, Maseda A, Lorenzo-López L, López-López R, Buján A, Rodríguez-Villamil JL (2018). Social factors and quality of life aspects on frailty syndrome in community-dwelling older adults: The VERISAÚDE study. BMC Geriatr.

[17] Yang YC, McClintock MK, Kozloski M, Li T (2013). Social isolation and adult mortality: the role of chronic inflammation and sex differences. J Health Soc Behav.

[18] Bloom I, Edwards M, Jameson KA, Syddall HE, Dennison E, Gale CR (2017). Influences on diet quality in older age: the importance of social factors. Age Ageing.

[19] Vesnaver E, Keller HH (2011). Social influences and eating behavior in later life: a review. J Nutr Gerontol Geriatr.

[20] Shankar A, McMunn A, Banks J, Steptoe A (2011). Loneliness, social isolation, and behavioral and biological health indicators in older adults. Heal Psychol.

[21] Saadeh M, Welmer A-K, Dekhtyar S, Fratiglioni L, Calderón-Larrañaga A. The role of psychological and social well-being on physical function trajectories in older adults. J Gerontol A Biol Sci Med Sci. 2020;75(8):1579–85. 10.1093/gerona/glaa114 PMC7357580 32384140

[22] Woo J (2015). Walking speed: a summary indicator of frailty?. J Am Med Dir Assoc.

[23] Pinheiro PA, Carneiro JAO, Coqueiro RS, Pereira R, Fernandes MH (2016). “Chair stand testˮ as simple tool for sarcopenia screening in elderly women. J Nutr Health Aging.

[24] Studenski S, Perera S, Patel K, Rosano C, Faulkner K, Inzitari M (2011). Gait speed and survival in older adults. JAMA.

[25] Ward RE, Leveille SG, Beauchamp MK, Travison T, Alexander N, Jette AM (2015). Functional performance as a predictor of injurious falls in older adults. J Am Geriatr Soc.

[26] Biessy C, Riboli E, Johansson I, Kaaks R (2002). Validation and calibration of food-frequency questionnaire measurements in the Northern Sweden Health and Disease cohort. Public Health Nutr.

[27] Shakersain B, Rizzuto D, Larsson S, Faxén-Irving G, Fratiglioni L, Xu W-L (2018). The nordic prudent diet reduces risk of cognitive decline in the swedish older adults: a population-based cohort study. Nutrients.

[28] Willett WC, Howe R (1997). Adjustment for total energy intake in epidemiologic studies. Am J Clin Nutr.

[29] Dohrn IM, Gardiner PA, Winkler E, Welmer AK, Welmer AK, Welmer AK (2020). Device-measured sedentary behavior and physical activity in older adults differ by demographic and health-related factors. Eur Rev Aging Phys Act.

[30] Calderón-Larrañaga A, Vetrano DL, Onder G, Gimeno-Feliu LA, Coscollar-Santaliestra C, Carfí A (2017). Assessing and measuring chronic multimorbidity in the older population: a proposal for its operationalization. J Gerontol - Ser A Biol Sci Med Sci.

[31] Milaneschi Y, Bandinelli S, Corsi AM, Lauretani F, Paolisso G, Dominguez LJ (2011). Mediterranean diet and mobility decline in older persons. Exp Gerontol.

[32] Talegawkar SA, Bandinelli S, Bandeen-Roche K, Chen P, Milaneschi Y, Tanaka T (2012). A higher adherence to a Mediterranean-style diet is inversely associated with the development of frailty in community-dwelling elderly men and women. J Nutr.

[33] Shahar DR, Houston DK, Hue TF, Lee JS, Sahyoun NR, Tylavsky FA (2012). Adherence to Mediterranean diet and decline in walking speed over 8 years in community-dwelling older adults. J Am Geriatr Soc.

[34] Struijk EA, Guallar-Castillón P, Rodríguez-Artalejo F, López-García E (2018). Mediterranean dietary patterns and impaired physical function in older adults. J Gerontol - Ser A Biol Sci Med Sci.

[35] León-Muñoz LM, Guallar-Castillón P, López-García E, Rodríguez-Artalejo F (2014). Mediterranean diet and risk of frailty in community-dwelling older adults. J Am Med Dir Assoc.

[36] Rahi B, Ajana S, Tabue-Teguo M, Dartigues JF, Peres K, Feart C (2018). High adherence to a Mediterranean diet and lower risk of frailty among French older adults community-dwellers: Results from the Three-City-Bordeaux Study. Clin Nutr.

[37] Romagnolo DF, Selmin OI (2017). Mediterranean diet and prevention of chronic diseases. Nutr Today.

[38] Granic A, Sayer AA, Robinson SM (2019). Dietary patterns, skeletal muscle health, and sarcopenia in older adults. Nutrients.

[39] Cesari M, Pahor M, Bartali B, Cherubini A, Penninx BWJH, Williams GR (2004). Antioxidants and physical performance in elderly persons: The Invecchiare in Chianti (InCHIANTI) study. Am J Clin Nutr.

[40] Chung HY, Cesari M, Anton S, Marzetti E, Giovannini S, Seo AY (2009). Molecular inflammation: underpinnings of aging and age-related diseases. Ageing Res Rev.

[41] Lauretani F, Russo CR, Bandinelli S, Bartali B, Cavazzini C, Di Iorio A (2003). Age-associated changes in skeletal muscles and their effect on mobility: an operational diagnosis of sarcopenia. J Appl Physiol.

[42] Grande G, Triolo F, Nuara A, Welmer AK, Fratiglioni L, Vetrano DL (2019). Measuring gait speed to better identify prodromal dementia. Exp Gerontol.

[43] McEvoy CT, Guyer H, Langa KM, Yaffe K (2017). Neuroprotective diets are associated with better cognitive function: the health and retirement study. J Am Geriatr Soc.

[44] GómezSánchez M, Gómez Sánchez L, Patino-Alonso MC, Alonso-Domínguez R, Sánchez-Aguadero N, Lugones-Sánchez C, et al. Adherence to the Mediterranean diet in Spanish population and its relationship with early vascular aging according to sex and age: EVA study. Nutrients. 2020;12(4):1025. 10.3390/nu12041025 PMC7231158 32276498

[45] Vassilaki M, Aakre JA, Syrjanen JA, Mielke MM, Geda YE, Kremers WK (2018). Mediterranean diet, its components, and amyloid imaging biomarkers. J Alzheimers Dis.

[46] Cesari F, Sofi F, Molino Lova R, Vannetti F, Pasquini G, Cecchi F (2018). Aging process, adherence to Mediterranean diet and nutritional status in a large cohort of nonagenarians: Effects on endothelial progenitor cells. Nutr Metab Cardiovasc Dis.

[47] Martínez-González MA, Gea A, Ruiz-Canela M (2019). The Mediterranean diet and cardiovascular health. Circ Res.

[48] Guasch-Ferré M, Merino J, Sun Q, Fitó M, Salas-Salvadó J (2017). Dietary polyphenols, mediterranean diet, prediabetes, and type 2 diabetes: a narrative review of the evidence. Oxid Med Cell Longev.

[49] Haider S, Grabovac I, Drgac D, Mogg C, Oberndorfer M, Dorner TE (2020). Impact of physical activity, protein intake and social network and their combination on the development of frailty. Eur J Public Health.

[50] Artaza-Artabe I, Sáez-López P, Sánchez-Hernández N, Fernández-Gutierrez N, Malafarina V (2016). The relationship between nutrition and frailty: Effects of protein intake, nutritional supplementation, vitamin D and exercise on muscle metabolism in the elderly. A systematic review. Maturitas.

[51] Kavouras SA, Panagiotakos DB, Pitsavos C, Chrysohoou C, Arnaoutis G, Skoumas Y, Stefanadis C. Physical activity and adherence to Mediterranean diet increase total antioxidant capacity: The ATTICA study. Cardiol Res Pract. 2010;2011:248626. 10.4061/2011/248626 PMC2963115 20981278

[52] Bell KE, Séguin C, Parise G, Baker SK, Phillips SM (2015). Day-to-day changes in muscle protein synthesis in recovery from resistance, aerobic, and high-intensity interval exercise in older men. J Gerontol Ser A Biol Sci Med Sci.

[53] Radak Z, Hart N, Sarga L, Koltai E, Atalay M, Ohno H (2010). Exercise plays a preventive role against Alzheimer’s disease. de la Torre JC, editor. J Alzheimer’s Dis..

[54] Cartee GD, Hepple RT, Bamman MM, Zierath JR (2017). Exercise promotes healthy aging of skeletal muscle primary versus secondary aging. Setting Stage..

[55] Sahyoun NR, Zhang XL, Serdula MK (2006). Barriers to the consumption of fruits and vegetables among older adults. J Nutr Elder.

[56] Locher JL, Ritchie CS, Robinson CO, Roth DL, West DS, Burgio KL (2008). A multidimensional approach to understanding under-eating in homebound older adults: the importance of social factors. Gerontologist.

[57] Thoits PA (2011). Mechanisms linking social ties and support to physical and mental health. J Health Soc Behav.

[58] Michels KB, Willett WC (2009). Self-Administered Semiquantitative Food Frequency Questionnaires. Epidemiology.

