# Supplementary figures and images for: Mobility and muscle strength trajectories in old age: the beneficial effect of Mediterranean diet in combination with physical activity and social support
Source: Int J Behav Nutr Phys Act. 2021 Sep 8;18:120. doi: 10.1186/s12966-021-01192-x (PMC8425101; doi:10.1186/s12966-021-01192-x)

**Additional file 1. Population flow-chart for baseline and follow-up assessments.**


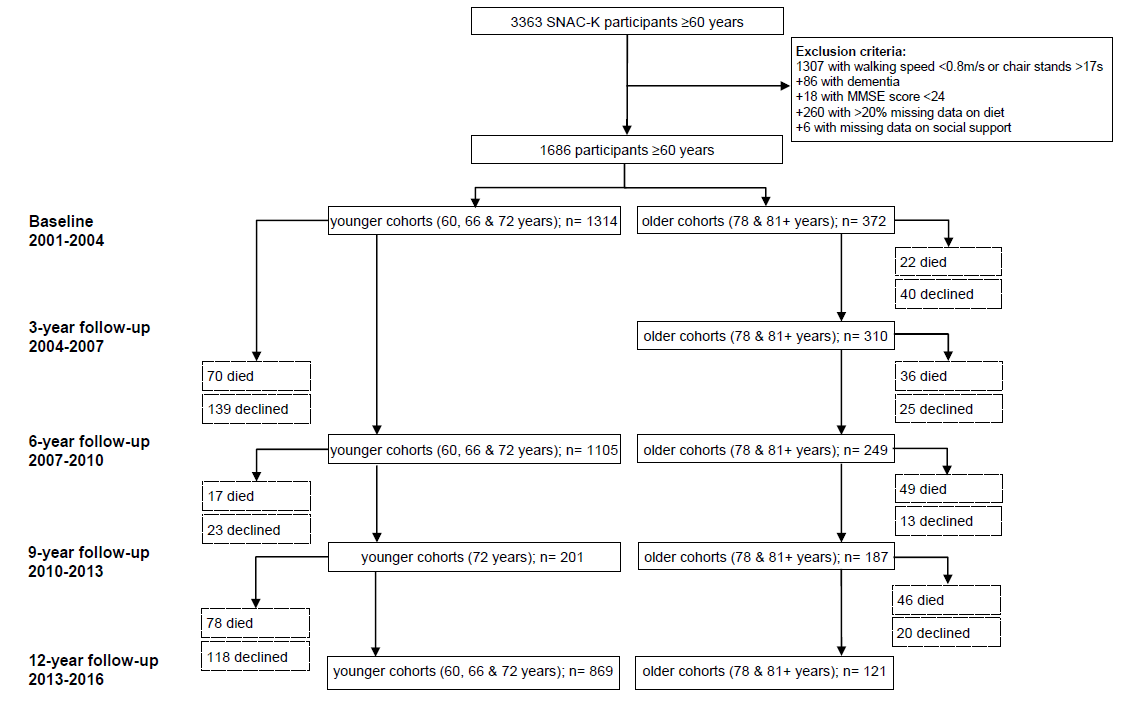

Supplement: Supplementary file 1 — Additional file 1. Population flow-chart for baseline and follow-up assessments. [file 12966_2021_1192_MOESM1_ESM.docx]
