# Supplementary material for: Mobility and muscle strength trajectories in old age: the beneficial effect of Mediterranean diet in combination with physical activity and social support
Source: Int J Behav Nutr Phys Act. 2021 Sep 8;18:120. doi: 10.1186/s12966-021-01192-x (PMC8425101; doi:10.1186/s12966-021-01192-x)
Supplement: Supplementary file 2 — Additional file 2. Dietary components of MDS and their cut-offs according to the median by sex. [file 12966_2021_1192_MOESM2_ESM.docx]

**Additional file 2. Dietary components of MDS and their cut-offs according to the median by sex.**

| **MDS items** | **Median (IQR) daily frequency** | | **Food items** | **% of missingness** |
| --- | --- | --- | --- | --- |
|  | **Male (n=714)** | **Female (n=972)** |  |  |
| Fish | 0.3 (0.2;0.4) | 0.3 (0.2;0.4) | Lean fish (e.g. bass, codfish, coalfish)  Fatty fish (e.g. herring, Baltic herring, whitefish, salmon, mackerel, eel)  Pickled fish (e.g. salt herring, Baltic herring)  Shellfish (e.g. shrimp, clam, crayfish) | 0.8  0.5  0.8  0.5 |
| Vegetables (excluding potatoes) | 2.1 (1.3;3.2) | 2.7 (1.8;3.8) | Tomato  Cucumber  Carrot  Root vegetables (e.g. turnip, parsnip);  White and red cabbage  Lettuce, Chinese cabbage  Spinach, kale or borecole  Cauliflower, Brussels sprouts, broccoli  Mushrooms  Frozen vegetable mixture  Vegetable casseroles  Vegetable gratin | 2.1  2.0  2.1  3.0  2.8  2.4  2.5  2.0  2.2  2.2  1.7  0.6 |
| Fruits | 1.2 (0.5;1.9) | 1.8 (1.1;2.7) | Berries (fresh or deep-frozen)  Apples, pears, peaches  Orange, mandarin, grapefruit  Banana | 4.0  2.7  2.5  2.4 |
| Legumes | 0.1 (0.1;0.2) | 0.1 (0.1;0.2) | Red beans, pea soup  Other beans soup | 0.8  1.0 |
| Grains | 3.2 (2.3;4.5) | 3.3 (2.4;4.3) | White bread  Thick soft whole grain bread  Hard bread, crisp hard-rye bread (e.g. Husmans)  Wheat crisp flat bread, light oven flat bread  Crackers  Porridge of oatmeal, rye or barley  Porridge of semolina or grain of rice  Gruel  Fibre-rich cereals (e.g. musli)  Corn flakes  Rice  Spaghetti, macaroni | 3.0  2.4  1.8  5.9  2.8  1.4  1.9  1.0  1.5  2.6  0.7  0.7 |
| Dairy products | 2.3 (1.4;3.4) | 2.6 (1.8;3.7) | Low-fat hard cheese 10-17%  Soft cheese, whey cheese  Low-fat milk 0.5%, 0.1%, low-fat sour milk  Light yoghurt, Hälsofil  Medium-fat hard cheese 28%  Medium-fat milk, medium-fat soured milk 1.5%  Cream, crème fraiche, sour cream  Full-cream cheese, dessert cheese  Standard-fat (normal) milk, soured milk 3%  Yoghurt, kefir | 5.5  2.6  4.9  6.3  2.5  3.4  2.6  2.3  3.0  4.0 |
| Meat (red/processed) | 1.3 (0.8;1.9) | 0.9 (0.6;1.5) | Minced meat (e.g. meatballs, hamburger, mincemeat sauce)  Meat casseroles  Whole meat (e.g. roast meat, cutlet)  Sauce for meat and fish dishes  Liver, kidney  Sausages cold cuts  Meat toppings  Liver pâté  Bacon, flitch of bacon  Sausage dish  Blood meal (e.g. blood sausage, blood pudding) | 0.6  0.8  0.3  0.8  0.8  1.6  2.1  1.0  0.7  0.4  0.5 |
| PUFA (g/day) | 10.8 (8.1;14.0) | 7.4 (5.6;9.6) | --- | 0.0 |
| MUFA (g/day) | 22.4 (17.1;29.1) | 15.5 (11.8;19.2) | --- | 0.0 |
| SAFA (g/day) | 24.6 (18.0;32.1) | 17.2 (12.8;22.5) | --- | 0.0 |
| Alcohol (g/day)* | 10–50 | 5–25 | --- | 0.3 |

MDS: Mediterranean Diet Score, IQR: interquartile range, PUFA: polyunsaturated fatty acids, MUFA: monounsaturated fatty acids, SAFA: saturated fatty acids

*For alcohol intake, cut-offs are defined according to pre-established ranges instead of sex-specific medians.
