# Supplementary material for: Mobility and muscle strength trajectories in old age: the beneficial effect of Mediterranean diet in combination with physical activity and social support
Source: Int J Behav Nutr Phys Act. 2021 Sep 8;18:120. doi: 10.1186/s12966-021-01192-x (PMC8425101; doi:10.1186/s12966-021-01192-x)
Supplement: Supplementary file 4 — Additional file 4. Baseline sociodemographic, clinical and lifestyle characteristics of the included and excluded population. [file 12966_2021_1192_MOESM4_ESM.docx]

**Additional file 4. Baseline sociodemographic, clinical and lifestyle characteristics of the included and excluded population.**

|  | **SNAC-K population (N=3363)** | **Analytical sample (n=1686)** | **Excluded individuals (n=1677)** |
| --- | --- | --- | --- |
| **Age, n(%)** |  |  |  |
| <78 years | 1780 (52.9) | 1314 (77.9) | 466 (27.8) |
| ≥78 years | 1574 (46.8) | 372 (22.1) | 1202 (71.7) |
| **Sex, n(%)** |  |  |  |
| Male | 1181 (35.1) | 714 (42.4) | 467 (27.9) |
| Female | 2182 (64.9) | 972 (67.7) | 1210 (72.2) |
| **Education, n(%)** |  |  |  |
| Elementary | 590 (17.5) | 152 (9.0) | 438 (26.1) |
| High school | 1651 (49.1) | 784 (46.5) | 867 (51.7) |
| University | 1090 (32.4) | 750 (44.5) | 340 (20.3) |
| **Civil status, n(%)** |  |  |  |
| Unmarried | 569 (16.9) | 275 (16.3) | 294 (17.5) |
| Married | 1444 (42.9) | 935 (55.5) | 509 (30.4) |
| Divorced | 439 (13.1) | 237 (14.1) | 202 (12.1) |
| Widow | 896 (26.6) | 237 (14.1) | 659 (39.3) |
| **Chronic diseases, median (IQR)** | 4 (2;5) | 3 (2;4) | 5 (3;6) |
| **Dietary supplements, n(%)** | |  |  |
| No | 2440 (72.6) | 1262 (74.9) | 1178 (70.2) |
| Yes | 923 (27.5) | 424 (25.2) | 499 (29.8) |
| **Social support (z-score), median (IQR)** | 0.1 (-0.3;0.4) | 0.2 (-0.1;0.5) | -0.4 (-0.5;0.3) |
| **Social support, n(%)** |  |  |  |
| Low | 1002 (29.8) | 562 (33.3) | 439 (26.2) |
| Moderate | 1003 (29.8) | 562 (33.3) | 436 (26.0) |
| High | 993 (29.5) | 562 (33.3) | 437 (26.1) |
| **Physical activity, n(%)** | |  |  |
| Inadequate | 1163 (34.6) | 263 (15.6) | 900 (53.7) |
| Health enhancing | 1537 (45.7) | 908 (53.9) | 629 (37.5) |
| Fitness enhancing | 663 (19.7) | 515 (30.6) | 148 (8.8) |
| **Mediterranean diet score, median (IQR)** | 2 (1;3) | 5 (3;6) | 1 (1;2) |
| **Adherence to Mediterranean diet, n(%)** | |  |  |
| Low | 1149 (34.2) | 802 (47.6) | 397 (23.7) |
| Moderate | 505 (15.0) | 365 (21.7) | 130 (7.8) |
| High | 700 (20.8) | 519 (30.8) | 141 (8.4) |
| **Total energy intake (Kcal/day),**  **median (IQR)** | 1581.0 (1251.4;1992.7) | 1601.6 (1274.6;1992.7) | 1552.4 (1204.4;1989.9) |
| **Walking speed (m/s)** |  |  |  |
| <0.8 | 970 (28.8) | - | 970 (57.8) |
| **≥**0.8 | 2271 (67.5) | 1669 (99) | 602 (35.9) |
| **Chair stands (s)** |  |  |  |
| <17 | 1918 (57.0) | 1565 (92.8) | 353 (21.1) |
| ≥17 | 515 (15.3) | - | 515 (30.7) |

Low, moderate and high levels of social support and adherence to Mediterranean diet categorized according to the tertiles of the distribution.

Levels of physical activity categorized as follows: inadequate (≤2–3 times per month of light and/or moderate/intense exercise), health-enhancing (light exercise several times per week or every day), and fitness-enhancing (moderate/intense exercise several times per week or every day).

Divergences between total numbers and the sample sizes is due to missing data.

IQR: interquartile range
