# Supplementary material for: Mobility and muscle strength trajectories in old age: the beneficial effect of Mediterranean diet in combination with physical activity and social support
Source: Int J Behav Nutr Phys Act. 2021 Sep 8;18:120. doi: 10.1186/s12966-021-01192-x (PMC8425101; doi:10.1186/s12966-021-01192-x)
Supplement: Supplementary file 5 — Additional file 5. Sensitivity analysis. Association between adherence to Mediterranean diet and annual decline in walking speed (m/s) and chair stands (s) over the 12-year follow-up (N=1686). Results after adjusting additionally by physical activity and social support. [file 12966_2021_1192_MOESM5_ESM.docx]

**Additional file 5. Sensitivity analysis. Association between adherence to Mediterranean diet and annual decline in walking speed (m/s) and chair stands (s) over the 12-year follow-up (N=1686). Results after adjusting additionally by physical activity and social support.**

|  | **β (95% CI)** | **p-value** |
| --- | --- | --- |
| **Walking speed (m/s)** |  |  |
| **Continuous** | 0.001 (0.0002;0.002) | **0.020** |
| **Categorical** |  |  |
| Low | Ref | Ref |
| Moderate | 0.003 (-0.002;0.007) | 0.307 |
| High | 0.006 (0.001;0.010) | **0.011** |
| **Chair stands (s)** |  |  |
| **Continuous** | -0.014 (-0.025;-0.004) | **0.008** |
| **Categorical** |  |  |
| Low | Ref | Ref |
| Moderate | -0.023 (-0.070;0.024) | 0.335 |
| High | -0.064 (-0.106;-0.023) | **0.002** |

Model adjusted by sex, age, education level, civil status, number chronic diseases at baseline, dietary supplements, death/dropouts, physical activity and social support.

Low, moderate and high levels of adherence to Mediterranean diet categorized according to the tertiles of the distribution.

CI: confidence interval
