# Supplementary material for: Mobility and muscle strength trajectories in old age: the beneficial effect of Mediterranean diet in combination with physical activity and social support
Source: Int J Behav Nutr Phys Act. 2021 Sep 8;18:120. doi: 10.1186/s12966-021-01192-x (PMC8425101; doi:10.1186/s12966-021-01192-x)
Supplement: Supplementary file 6 — Additional file 6. Sensitivity analysis. Association between adherence to Mediterranean diet and annual decline in walking speed (m/s) and chair stands (s) over the 12-year follow-up. Results after excluding participants with less than two measures of walking speed (m/s) or chair stands (s). [file 12966_2021_1192_MOESM6_ESM.docx]

**Additional file 6. Sensitivity analysis. Association between adherence to Mediterranean diet and annual decline in walking speed (m/s) and chair stands (s) over the 12-year follow-up. Results after excluding participants with less than two measures of walking speed (m/s) or chair stands (s).**

|  | **Model I** | | **Model II** | |
| --- | --- | --- | --- | --- |
|  | **β (95% CI)** | **p-value** | **β (95% CI)** | **p-value** |
| **Walking speed (m/s)**  **(n=1409)** |  |  |  |  |
| **Continuous** | 0.001 (0.00001;0.002) | **0.048** | 0.001 (0.0001;0.002) | **0.040** |
| **Categorical** |  |  |  |  |
| Low | Ref | Ref | Ref | Ref |
| Moderate | 0.002 (-0.003;0.007) | 0.507 | 0.002 (-0.003;0.007) | 0.448 |
| High | 0.005 (0.0004;0.009) | **0.033** | 0.005 (0.001; 0.009) | **0.027** |
| **Chair stands (s)**  **(n=1255)** |  |  |  |  |
| **Continuous** | -0.015 (-0.026;-0.004) | **0.007** | -0.015 (-0.025;-0.004) | **0.007** |
| **Categorical** |  |  |  |  |
| Low | Ref | Ref | Ref | Ref |
| Moderate | -0.021 (-0.069;0.027) | 0.392 | -0.020 (-0.068;0.027) | 0.404 |
| High | -0.068 (-0.110;-0.026) | **0.002** | -0.067 (-0.109;-0.025) | **0.002** |

Model I: adjusted by sex, age, education level.

Model II: adjusted additionally by civil status, number chronic diseases at baseline, dietary supplements and death/dropouts.

Low, moderate and high levels of adherence to Mediterranean diet categorized according to the tertiles of the distribution.

CI: confidence interval
