# Supplementary material for: Mobility and muscle strength trajectories in old age: the beneficial effect of Mediterranean diet in combination with physical activity and social support
Source: Int J Behav Nutr Phys Act. 2021 Sep 8;18:120. doi: 10.1186/s12966-021-01192-x (PMC8425101; doi:10.1186/s12966-021-01192-x)
Supplement: Supplementary file 7 — Additional file 7. Sensitivity analysis. Association between adherence to Mediterranean diet and annual decline in walking speed (m/s) and chair stands (s) over the 12-year follow-up (N=892). Results after excluding participants with any missing data on the food items comprising the MDS (i.e. complete case analysis). [file 12966_2021_1192_MOESM7_ESM.docx]

**Additional file 7. Sensitivity analysis. Association between adherence to Mediterranean diet and annual decline in walking speed (m/s) and chair stands (s) over the 12-year follow-up (N=892). Results after excluding participants** **with any missing data on the food items comprising the MDS (i.e. complete case analysis).**

|  | **Model I** | | **Model II** | |
| --- | --- | --- | --- | --- |
|  | **β (95% CI)** | **p-value** | **β (95% CI)** | **p-value** |
| **Walking speed (m/s)** |  |  |  |  |
| **Continuous** | 0.002 (0.0001;0.003) | **0.037** | 0.002 (0.0002;0.003) | **0.026** |
| **Categorical** |  |  |  |  |
| Low | Ref | Ref | Ref | Ref |
| Moderate | 0.001 (-0.006;0.007) | 0.859 | 0.001 (-0.006;0.008) | 0.750 |
| High | 0.006 (-0.00004;0.012) | **0.051** | 0.006 (0.0003; 0.012) | **0.039** |
| **Chair stands (s)** |  |  |  |  |
| **Continuous** | -0.025 (-0.039;-0.010) | **0.001** | -0.025 (-0.040 -0.011) | **0.001** |
| **Categorical** |  |  |  |  |
| Low | Ref | Ref | Ref | Ref |
| Moderate | -0.070 (-0.13;0.005) | **0.035** | -0.072 (-0.137;-0.007) | **0.029** |
| High | -0.106 (-0.163;-0.050) | **<0.001** | -0.108 (-0.165;-0.052) | **<0.001** |

Model I: adjusted by sex, age, education level.

Model II: adjusted additionally by civil status, number chronic diseases at baseline, dietary supplements and death/dropouts

Low, moderate and high levels of adherence to Mediterranean diet categorized according to the tertiles of the distribution.

CI: confidence interval
