# Supplementary material for: Mobility and muscle strength trajectories in old age: the beneficial effect of Mediterranean diet in combination with physical activity and social support
Source: Int J Behav Nutr Phys Act. 2021 Sep 8;18:120. doi: 10.1186/s12966-021-01192-x (PMC8425101; doi:10.1186/s12966-021-01192-x)
Supplement: Supplementary file 8 — Additional file 8. Association between levels of adherence to Mediterranean diet and annual decline in walking speed (m/s) and chair stands (seconds) over the 12-year follow-up, by levels of social support and physical activity (N=1686). [file 12966_2021_1192_MOESM8_ESM.docx]

**Additional file 8. Association between levels of adherence to Mediterranean diet and annual decline in walking speed (m/s) and chair stands (seconds) over the 12-year follow-up, by levels of social support and physical activity (N=1686).**

|  | **β (95% CI)** | **p-value** |
| --- | --- | --- |
| **Indicator variable for MDS and social support** |  |  |
| **Walking speed (m/s)** |  |  |
| Low adherence, Low social support | Ref |  |
| Low adherence, High social support | 0.004 (-0.002;0.010) | 0.189 |
| High adherence, Low social support | 0.007 (-0.0003;0.013) | 0.062 |
| High adherence, High social support | 0.007 (0.001;0.013) | **0.014** |
| **Chair stands (s)** |  |  |
| Low adherence, Low social support | Ref |  |
| Low adherence, High social support | -0.016 (-0.076;0.043) | 0.597 |
| High adherence, Low social support | -0.045 (-0.11;0.023) | 0.197 |
| High adherence, High social support | -0.065 (-0.012;-0.008) | **0.026** |
| **Indicator variable for MDS and physical activity** |  |  |
| **Walking speed (m/s)** |  |  |
| Low adherence, Low physical activity | Ref |  |
| Low adherence, High physical activity | -0.0004 (-0.006;0.007) | 0.901 |
| High adherence, Low physical activity | 0.002 (-0.003;-0.007) | 0.469 |
| High adherence, High physical activity | 0.010 (0.004;0.015) | **0.001** |
| **Chair stands (s)** |  |  |
| Low adherence, Low physical activity | Ref |  |
| Low adherence, High physical activity | -0.029 (-0.088;0.030) | 0.336 |
| High adherence, Low physical activity | -0.060 (-0.105;-0.014) | **0.011** |
| High adherence, High physical activity | -0.050 (-0.104;-0.002) | **0.041** |

Model adjusted by sex, age, education level, civil status, number chronic diseases at baseline, dietary supplements and death/dropouts.

Low and high levels of MD adherence categorized according to the median of the distribution; low and high (i.e. moderate/high) levels of social support according to the tertiles of the distribution, and low (i.e. inadequate and health-enhancing) and high (i.e. fitness-enhancing) levels of physical activity according to official recommendation, as described in the methods.

MDS: Mediterranean diet score; CI: confidence interval

Three-way interactions for walking speed as the outcome:

Mediterranean diet*time*social support (β= 0.00001; p=0.990); Mediterranean diet*time*physical activity (β=0.001; p=0.378)

Three-way interactions for chair stands as the outcome:

Mediterranean diet*time*social support (β= -0.005; p=0.654); Mediterranean diet*time*physical activity (β= 0.002; p=0.883)
